# Dupilumab Improves Facial Pain and Reduces Rescue Treatments in Patients with CRSwNP and Recalcitrant Frontal Sinusitis

**DOI:** 10.3390/jpm14070735

**Published:** 2024-07-09

**Authors:** Eugenio De Corso, Stefano Settimi, Daniele Penazzi, Giuseppe D’Agostino, Marco Corbò, Mario Rigante, Claudio Montuori, Alberta Rizzuti, Maria Clara Pacilli, Tiziana Di Cesare, Simone Lo Verde, Angela Rizzi, Raffaella Chini, Jacopo Galli

**Affiliations:** 1 Unit of Otorhinolaryngology and Head-Neck Surgery, “A. Gemelli” University Hospital Foundation IRCCS, 00168 Rome, Italy; eugenio.decorso@policlinicogemelli.it (E.D.C.); mario.rigante@policlinicogemelli.it (M.R.); tiziana.dicesare@guest.policlinicogemelli.it (T.D.C.); jacopo.galli@unicatt.it (J.G.); 2 Department of Head-Neck and Sensory Organs, Catholic University of Sacred Heart, 00168 Rome, Italy; daniele.penazzi01@icatt.it (D.P.); giuseppe.dagostino01@icatt.it (G.D.); marco.corbo@guest.policlinicogemelli.it (M.C.); claudio.montuori01@icatt.it (C.M.); alberta.rizzuti@guest.policlinicogemelli.it (A.R.); mariaclara.pacilli01@icatt.it (M.C.P.); simone.loverde01@icatt.it (S.L.V.); 3 Unit of Allergology e Clinical Immunology, “A. Gemelli” Hospital Foundation IRCCS, 00168 Rome, Italy; angela.rizzi@policlinicogemelli.it (A.R.); raffaella.chini@policlinicogemelli.it (R.C.)

**Keywords:** chronic rhinosinusitis, nasal polyps, biologics, endoscopic sinus surgery, frontal sinusitis, headache, oral corticosteroids

## Abstract

Recalcitrant frontal sinusitis in patients with chronic rhinosinusitis and nasal polyps (CRSwNP) has a negative impact on their quality of life due to frontal pain and a high risk of sinus occlusion, thus necessitating antibiotics, systemic corticosteroids, and multiple surgeries. The aim of this study was to assess the efficacy of dupilumab in reducing frontal pain and the need for rescue treatments for recalcitrant frontal sinusitis in patients with CRSwNP. We enrolled a cohort of 10 patients with severe uncontrolled CRSwNP and concomitant recurrent frontal sinusitis associated with severe facial pain measured by MIDAS score who were treated with dupilumab 300 mg every 2 weeks and followed for at least 12 months. The mean MIDAS score decreased from 45.6 ± 10.7 at baseline to 1.3 ± 2.3 at 6 months (*p* < 0.05). VAS craniofacial pain decreased from 7.3 ± 1.6 at baseline to 1.2 ± 1.5 at 6 months (*p* < 0.05). No patient needed oral corticosteroids during treatment with dupilumab (*p* < 0.05), and the use of analgesics decreased from 9.6 ± 3.1 NSAID pills/week in the last 2 months at baseline to 0.6 ± 1.3 at 1 year of follow-up (*p* < 0.05). Our results demonstrated that use of subcutaneous dupilumab can improve symptom control, including recurrent severe cranio-facial pain, and reduce the need for rescue medical treatments (systemic steroids and NSAID) in patients with severe uncontrolled CRSwNP and concomitant recurrent frontal sinusitis.

## 1. Introduction

Severe uncontrolled chronic rhinosinusitis with nasal polyps (CRSwNP) has a significant negative impact on the quality of life of patients [1,2,3]. In the majority of cases, clinical manifestations are caused and sustained by a predominant type 2 inflammatory profile, characterized by the presence of interleukins IL-4, IL-5, and IL-13 as well as an inflammatory infiltrate characterized by the presence of eosinophils, basophils, and mast cells. At sites of inflammation, inflammatory mediators contribute to the pathogenesis of the disease, leading to excessive tissue remodeling. In particular, IL-13 is thought to be a key driver of airway epithelial remodeling and cell-type compositional changes [4,5]. Recalcitrant frontal sinusitis may be associated with severe uncontrolled CRSwNP and further increases the burden of the disease on the quality of life, which in some patients may be debilitating. Indeed, recurrent frontal sinusitis may worsen symptoms, especially frontal pain, increasing the need for courses of systemic corticosteroids, non-steroidal anti-inflammatory drugs (NSAIDs), and multiple surgeries to avoid sinus occlusion and complications. Unfortunately, even if the surgery is effective and radical, re-stenosis of the frontal ostium often occurs and can represent a persistent problem [6]. This increases the risk of recurrent frontal sinusitis due to bacterial superinfections, which requires the prolonged use of antibiotics and, in some cases, a new surgery to prevent major complications.

Therefore, in patients with severe uncontrolled CRSwNP and concomitant recurrent frontal sinusitis, it may be possible to obtain some benefits with biologics, especially considering that dupilumab has been demonstrated to be very effective on both the inflammatory response and tissue remodeling by inhibiting the signaling of IL-4 and IL-13. Indeed, its efficacy in severe uncontrolled CRSwNP was demonstrated in the LIBERTY NP SINUS trials [7], where it was associated with significant improvement (starting from the fourth week of treatment) in all primary and secondary endpoints (nasal congestion/obstruction severity, nasal polyp score, sinus opacification, and loss of smell) at weeks 24 and 52 of treatment. The effectiveness and safety of dupilumab were further demonstrated in real-life studies [8] for all the most important outcomes (reducing polyp size, improving the quality of life, reducing the severity of symptoms, resolving nasal congestion, improving smell, reducing the need for surgery, and/or oral corticosteroids) irrespective of previous treatments (surgery or other biologics) and coexisting comorbidities [9].

The objective of the present study was to evaluate the benefits of dupilumab in patients with CRSwNP and concomitant recalcitrant frontal sinusitis. In particular, we aimed to verify its efficacy in reducing sinonasal symptoms including severe frontal pain and in reducing the need for rescue treatments. 

## 2. Materials and Methods

### 2.1. Study Population, Inclusion Criteria, and Exclusion Criteria

This was a real-life, observational, non-profit case series study on patients with severe CRSwNP and concomitant recurrent frontal sinusitis who were complaining of significant craniofacial pain and started treatment with dupilumab in real life between November 2022 and December 2023. We specifically enrolled patients who were complaining of significant craniofacial pain due to recalcitrant frontal sinusitis with at least one sinus completely opacified at computed tomography (CT) and who were candidates for revision frontal sinusotomy.

Inclusion criteria were age ≥ 18 years; dupilumab prescribed for severe uncontrolled CRSwNP based on the indications of the Italian Agency of Drugs (AIFA) (diffuse CRSwNP confirmed by endoscopy and CT who met the following criteria: severe disease stage (NPS ≥ 5 and/or SNOT-22 ≥ 50); inadequate symptom control with intranasal corticosteroids (INCS); failure (or intolerance) of previous medical treatments (at least 2 cycles of systemic corticosteroids over the last year); and/or of previous endoscopic sinus surgery (ESS)) [1,9]; significant frontal facial pain in the last 3 months; VAS facial pain > 5 at the baseline; severe disability measured by MIDAS score > 20; high need for NSAIDs (>5 pills per week in the last 2 months); and complete opacification of at least one frontal sinus at baseline CT scan. 

Exclusion criteria: secondary CRS (cystic fibrosis, sinonasal tumor, primary ciliary dyskinesia, or autoimmune disease); continuous systemic steroid treatment; sinonasal granulomatous disease/tumor; previous radiotherapy for head and neck cancer; complication of frontal sinusitis for which surgery was necessary. 

Dupilumab 300 mg was self-administered subcutaneously every two weeks as add-on therapy to INCS. The study was approved by the local ethics committee (Protocol ID-4429). Informed consent about privacy and utilization of clinical data was obtained from all patients at the time of the original data collection; clinical data were therefore anonymously analyzed.

### 2.2. Study Design and Outcomes

Data were retrospectively analyzed at baseline and at 3, 6, and 12 months of follow-up. We took into consideration clinical data such as patient demographics, use of previous biologics, number and type of previous surgeries, previous use of anti-inflammatory drugs or analgesics, and use of oral corticosteroids in the previous year. We analyzed the response to treatment during the first year of follow-up: for this purpose, we took into consideration CRSwNP-related outcomes (NPS, NCS, SNOT-22, Sniffin’ Sticks, VAS for nasal symptoms, nasal cytology, and need for short course of OCS) and craniofacial pain-related measures (VAS craniofacial pain, MIDAS score, need for analgesics, and EQ-VAS). The specific outcomes measured were:

- Sinonasal Outcome Test (SNOT)-22: we utilized the validated Italian version of the SNOT-22 questionnaire for the evaluation of quality of life [10].
- Nasal endoscopy with nasal polyp score (NPS): each side of the nasal cavity was separately evaluated and scored according to the last EAACI position paper [11]; each side of the nasal cavity was separately evaluated and scored in a range from 0 to 4 (0 = no polyps, 1 = small polyps in the middle meatus not reaching below the inferior border of the middle turbinate, 2 = polyps reaching below the lower border of the middle turbinate, 3 = large polyps reaching the lower border of the inferior turbinate or polyps medial to the middle turbinate, and 4 = large polyps causing complete obstruction of the inferior nasal cavity). The sum of scores for both nasal cavities was recorded as the NPS value.
- Nasal Congestion Score (NCS): patients evaluated their symptoms of congestion/obstruction from the previous day using the NC scale (0: no symptoms; 1: mild symptoms (symptoms clearly present, but minimal awareness and easily tolerated); 2: moderate symptoms (definite awareness of symptoms that are bothersome but tolerable); 3: severe symptoms (symptoms that are hard to tolerate and cause interference with activities of daily living)) [12].
- VAS for nasal symptoms: the intensity of symptoms (nasal obstruction, rhinorrhea, smell, cranio-facial pain) was assessed using a horizontal 10 cm line with points from 0 (no symptom at all) to 10 (symptom completely debilitating) [11].
- EQ-5D-5L: we specifically used the EQ-VAS, which records the respondent’s overall current health on in a vertical visual analogue scale from 0 to 100 points, where the endpoints are labelled “The best health you can imagine” (100 points) and “The worst health you can imagine” (0 points). The EQ-VAS provides a quantitative measure of the patients’ perception of their overall health [13].
- Sniffin’ Sticks 16-identification test to assess the olfactory function: this test is performed by administering 16 odors at suprathreshold intensity to the patient. Patients must identify each odor presented by choosing from the four options provided. Depending on the number of correctly identified substances, a result between 0 (no substance identified) and 16 (all substances identified) is obtained. This allowed us to classify patients as anosmic (score between 0 and 5), hyposmia (score between 6 and 10) or normosmic (score major than 11 until 16) [14,15].
- Migraine Disability Assessment score (MIDAS): this questionnaire assesses the level of disability caused by migraines over a period of 3 months. It is a self-administered questionnaire that provides a quantitative measure of headache-related disability, assessing the amount of time lost for schoolwork or work; household work or chores; and family, social, and leisure activities. The scores obtained with the MIDAS questionnaire have a strong correlation with physician assessments of the severity of illness and the need for treatment. The scoring system for the MIDAS questionnaire is as follows: 5 to 10 indicates little or no disability; 10 to 20 indicates moderate disability; and a score higher than 20 denotes severe disability [16].
- We gathered information on the use of NSAIDs and systemic steroids both pre- and post-treatment. Need for systemic steroids was assessed considering the number of brief cycles per year. The need for NSAIDs was measured by the mean number of pills per week in the last 2 months.
- Nasal cytology was used to evaluate the presence of local eosinophilic inflammation: the examination was carried out on the material taken from the lower and middle turbinate bilaterally by “scraping” the mucosa with a Rhino-probe (Farmark SNC, Milan, Italy). The sample was gently spread on glass slides and immediately fixed in 95% ethyl alcohol and stained with May–Grunwald–Giemsa. The slides were examined under oil immersion by light microscopy first at a magnification of 400× and then at a magnification of 1000×. Nasal tissue eosinophil infiltration was measured as “eosinophil count per high power field (Ec-hpf)” and reported as the mean of at least three of the richest high-power fields observed at nasal cytology [17].

### 2.3. Statistical Analysis

Data were analyzed using SPSS 25 (IBM SPSS Statistics for Windows, Version 25.0. Armonk, NY, USA: IBM Corp.). Continuous data were tested for normality using the Kolmogorov–Smirnov test. Normally distributed data were described as mean ± standard deviation (SD) and compared using paired Student’s t-test for repeated measures. The significance threshold was set at *p* < 0.05.

## 3. Results

### 3.1. Baseline Characteristics of the Cohort

We enrolled a cohort of 10 consecutive patients (six females and four males; mean age: 56.1 ± 12.7 years, range 29–67). The mean number of previous endoscopic sinus surgeries for CRSwNP before starting dupilumab was 3.0 ± 1.9. All patients had significantly used OCS or NSAIDs for concomitant frontal recurrent sinusitis. More specifically, in the entire cohort, we documented a mean of 3.6 ± 1.4 brief cycles of OCS in the previous year and a mean of 9.6 ± 3.1 NSAID pills/week in the previous 2 months. Eight of the ten patients (80%) had comorbid asthma that was under control via inhalers. Four patients switched to dupilumab from another biologic: one patient from omalizumab, two from mepolizumab, and one from benralizumab because of inadequate control of sinonasal symptoms. All patients were on treatment with (mometasone furoate, 200 mcg per day) at baseline and continued the INCS as add-on therapy to dupilumab. In none of the patients was the dose or interval of dupilumab modified. Their socio-demographic and clinical characteristics are summarized in Table 1 and reported in detail in Table 2.

### 3.2. Changes in Sinonasal Outcomes

Regarding sinonasal outcomes, there was significant improvement within the first year of treatment with dupilumab for all outcomes considered. The NPS decreased from a mean of 3.1 ± 1.7 at baseline to 1.9 ± 1.2 at 3 months (*p* > 0.05), to 1.3 ± 1.2 at 6 months (*p* < 0.05), and to 0.2 ± 0.4 at 12 months (*p* < 0.05). Mean NCS values decreased from 2.6 ± 0.5 at baseline to 1.3 ± 0.8 at 3 months (*p* < 0.05) and to 1.1 ± 0.7 and 0.4 ± 0.5 at 6 and 12 months, respectively (*p* < 0.05).

Regarding quality of life, the mean SNOT-22 total score significantly decreased from 66.9 ± 11.2 to 40.3 ± 11.1 after 3 months of therapy (*p* < 0.05). This trend was sustained at 6 and 12 months, with values that decreased to 35.6 ± 7.4 and 20.6 ± 6.2, respectively (*p* < 0.05). The mean VAS score for nasal obstruction decreased from 7.0 ± 1.3 at baseline to 3.6 ± 1.8 at 3 months (*p* < 0.05), to 2.4 ± 2.0 at 6 months (*p* < 0.05), and to 1.3 ± 1.1 at 12 months (*p* < 0.05). Mean VAS rhinorrhea values decreased from 6.7 ± 1.4 at baseline to 2.9 ± 2.1 at 3 months (*p* < 0.05), to 2.6 ± 1.9 at 6 months (*p* < 0.05), and to 1.0 ± 1.1 at 12 months (*p* < 0.05).

For olfaction, the mean VAS smell values decreased from 7.1 ± 2.3 at baseline to 4.0 ± 1.8 at 3 months with no significant difference; however, mean values significantly decreased to 2.4 ± 1.6 at 6 months (*p* < 0.05) and to 1.4 ± 2.5 at 12 months (*p* < 0.05). Moreover, the Sniffin’ Sticks identification test total scores increased from a mean of 5.3 ± 3.0 at baseline to 7.1 ± 1.1 at 3 months (*p* < 0.05), 9.3 ± 1.6 at 6 months (*p* < 0.05), and 10.6 ± 1.5 at 12 months (*p* < 0.05).

Finally, a significant difference between local eosinophilia at nasal cytology before and after treatment was observed in all patients. Specifically, the mean Ec-hpf decreased from a mean of 30.9 ± 10.2 to 5.5 ± 3.5 (*p* < 0.05) after 12 months. 

### 3.3. Efficacy on Craniofacial Algia and Need for Analgesics

In all patients, we observed a significant reduction of cranio-frontal pain measured by MIDAS score and VAS craniofacial pain (Table 3). Analyzing the overall trend, we observed a significant reduction in the mean MIDAS score from 45.6 ± 10.7 at baseline to 10.43 ± 2.57 at 3 months (*p* < 0.05), 1.3 ± 2.3 at 6 months (*p* < 0.05) and 0.9 ± 0.6 at 12 months (*p* < 0.05). The same trend was observed for VAS craniofacial pain: this decreased from 7.3 ± 1.6 at baseline to 2.5 ± 3.2 at 3 months (*p* < 0.05), as well as 1.2 ± 1.5 and 1.0 ± 1.4 at 6 and 12 months, respectively (*p* < 0.05). 

**Figure 1:**
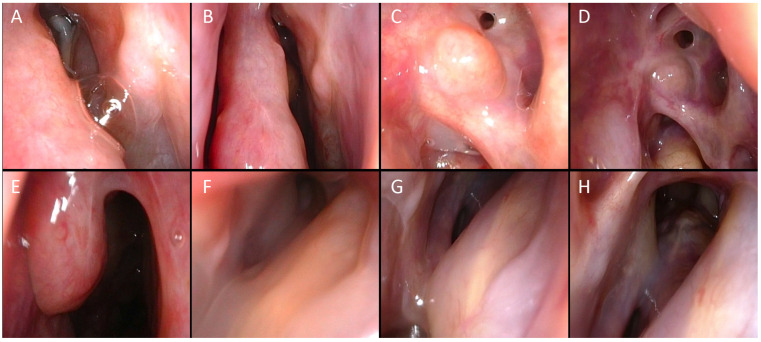
A 56-year-old man with 2 previous endoscopic sinus surgeries (1 ESS in 2016 and ESS + Draft 3 in 2019). One major complication during the last surgery (skull base CSF leak in ethmoid that was intraoperatively repaired). Recurrence of NP diagnosed in March 2022 with re-stenosis of the left frontal sinus with severe facial pain. Complete disappearance of polyps after three months of dupilumab administration with resolution of facial pain after 3 months. (**A**) Endoscopic view of the left osteomeatal complex with preserved middle turbinate. (**B**) Endoscopic view after 1 month of treatment. (**C**) Left frontal recess after 2 months of treatment. (**D**) Left frontal recess after 3 months of treatment. (**E**) Residual right middle turbinate. (**F**) Right frontal recess after 1 month of treatment. (**G**) Right frontal recess after 2 months of treatment. (**H**) Right frontal recess after 3 months of treatment.

**Figure 2:**
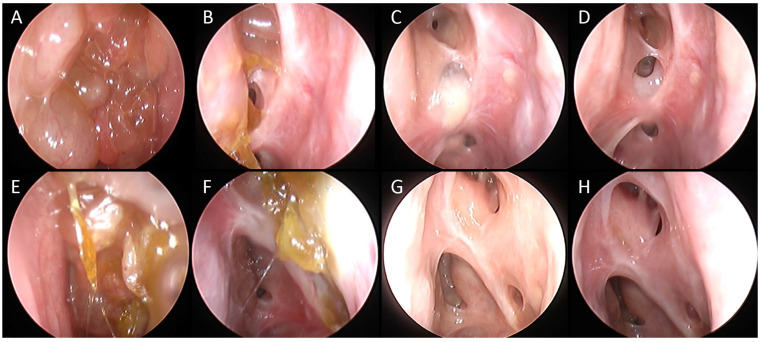
A 53-year-old man with medical history of multiple long-lasting cycles of oral corticosteroid in the last few years (>60 cumulative days/year) and previous treatment with mepolizumab. The patient had undergone six previous surgeries including a bilateral Draft 2b. In April 2022, recurrence of NP associated with severe facial pain was diagnosed. Complete resolution of pain and disappearance of polyps after 6 months of treatment with dupilumab. (**A**) Recurrence of nasal polyps in left frontal recess. (**B**) Left frontal recess view after 3 months of treatment. (**C**) Left frontal recess view after 6 months of treatment. (**D**) Left frontal recess view after 12 months of treatment. (**E**) Recurrence of nasal polyps in right frontal recess. (**F**) Right frontal recess view after 3 months of treatment. (**G**) Right frontal recess view after 6 months of treatment. (**H**) Right frontal recess view after 12 months of treatment.

**Figure 3:**
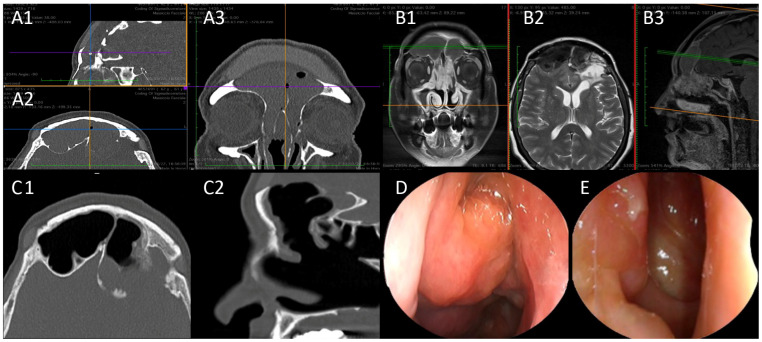
A 62-year-old woman with NSAID-ERD and medical history of multiple brief cycles of OCS in the last few years (>52 cumulative days/year). The patient had recalcitrant frontal sinusitis associated with invalidating frontal pain. Two previous surgeries including an external bicoronal approach to the frontal sinus and reconstruction with custom-made prosthesis. Diagnosis of recurrence of nasal polyps and mucocele was made in January 2023. After 12 months of dupilumab, the mucocele and nasal polyps had resolved. Herein we report the results after 12 months of treatment. (**A1**–**A3**) Pre-treatment CT-SCAN, complete opacification of the frontal sinuses ((**A1**) Sagittal view. (**A2**) Axial view. (**A3**) Coronal view). (**B1**–**B3**) Pre-treatment MRI showing recurrence of the nasal polyposis and frontal mucocele ((**B1**) Coronal view. (**B2**) Axial view. (**B3**) Sagittal view). (**C1**,**C2**) Post-treatment CT showing pneumatization of the frontal sinuses ((**C1**) Axial view. (**C2**) Sagittal view). (**D**) Pre-treatment endoscopic view. (**E**) Post-treatment endoscopic view.

Finally, we observed a significant reduction in terms of the rescue treatment needed, including the administration of OCS and analgesics (Table 3). Indeed, no patient needed a cycle of OCS during treatment with dupilumab (*p* < 0.05), and the use of analgesics decreased from 9.6 ± 3.1 pills/week in the last 2 months to 0.6 ± 1.3 pills/week during the last 2 months at 1 year of follow-up (*p* < 0.05). Finally, regarding the globally evaluated quality of life, the EQ-VAS improved from 50 ± 12.9 at baseline to 69.3 ± 11.3 at 3 months (*p* < 0.05) to 77.0 ± 14.2 and 81.4 ± 9.4 at 6 and 12 months of follow-up, respectively (*p* < 0.05). The most representative results after 6 months of treatment are reported in Table 3, while Figure 1, Figure 2 and Figure 3 show some of the most representative clinical cases and their outcomes after therapy with dupilumab.

## 4. Discussion

Recalcitrant frontal chronic sinusitis can occur in patients with severe CRSwNP, especially after multiple/inadequate surgeries [18], which is due to chronic sinus inflammation and abnormal scarring [19,20], and leads to an additional need for systemic treatments (corticosteroids and antibiotics) to manage exacerbations of symptoms and to treat associated severe craniofacial pain. 

In this report, we studied a very specific group of patients with recurrent frontal sinusitis in the context of uncontrolled severe CRSwNP. The co-existence of frontal sinusitis represents an additional burden in terms of the deterioration of patients’ quality of life and, in particular, in the presence of associated severe facial pain. In these patients, a surgical approach to the frontal sinus in CRSwNP may be required to restore frontal sinus ventilation and drainage. However, this kind of surgery can be challenging, especially in cases who underwent a previous surgery and are thus at higher risk for complications. The frontal recess is, in fact, located in an anterosuperior position, making endoscopic visualization and dissection difficult. Furthermore, the anatomy of the frontal recess and frontal ostium are quite complex, making any mucosal damage or inflammation resulting from surgery a significant concern. This complexity is heightened in cases of revision after multiple frontal sinus surgeries due to prior mucosal trauma and risk of fibrosis and stenosis, which frequently lead to the onset of recurrent sinusitis, associated with pain and the need for multiple interventions to re-open the frontal sinus. Furthermore, the frontal revision surgery may be even more challenging for the ENT surgeon in cases of an incomplete initial surgery, a lack of compliance with long-term post-operative local corticosteroids, or in the presence of predictors of severity (coexisting asthma, aspirin-exacerbated respiratory disease, and high inflammatory load). 

Over the years, surgeons have given significant value to the surgical clearance of the frontal recess [21]. For this reason, frontal sinus surgery was often extended, especially when standard sinus surgery had failed, resulting in stenosis of the frontal ostium due to scar tissue or new bone formation. Previous studies have shown that the primary Draf 3 procedure in patients with CRSwNP has a failure rate of 8.9%, while revision Draf 3 has a failure rate of 21% [22]. Furthermore, the success rates of the Draf 3 procedure may vary depending on the surgical technique and patient population such as NSAID-ERD [23]. While some authors have suggested that an extended procedure can solve the problem of recurrent frontal sinusitis [4,18,24], other authors avoid re-stenosis of the frontal sinus and have proposed mucosal flaps [23], balloon dilation, or local stents with a slow release of drugs [25]. In particular, recent advances in biomaterial technology have led to the development of corticosteroid-coated sinus stents, which can elute local corticosteroids, such as mometasone furoate, in a controlled manner via a bioabsorbable core. The stenting action is crucial in achieving this goal to separate the raw edges of the mucosal wound surface and to prevent the formation of an adhesion and stenosis. Two randomized controlled trials [26,27] have shown that bioabsorbable steroid-eluting stents may maintain the patency of the frontal sinuses and improve mucosal wound healing after surgery. However, very few studies have evaluated the use of bioabsorbable steroid-eluting sinus stents for the treatment of recalcitrant chronic frontal rhinosinusitis, and there is a lack of studies comparing the outcomes of eluting stents and biologics.

The advent of biologics has significantly changed the approach to patients with severe and uncontrolled CRSwNP. In particular, dupilumab is a promising therapeutic option in such patients, particularly those with type 2 inflammation. Its ability to target key cytokines involved in the inflammatory process not only helps to reduce polyp size and improve symptoms, but also affects the underlying tissue remodeling, leading to better clinical outcomes. 

We therefore tried to verify the hypothesis that dupilumab may offer some advantages in the management of patients with frontal recurrent sinusitis in severe CRSwNP. For the first time, we preliminarily demonstrated in a small cohort of these patients that dupilumab was effective in improving sinonasal outcomes and in reducing severe pain and the use of rescue treatments. This can be explained by its action in blocking type 2 inflammation by IL-4 as well as in reducing remodeling/fibrotic processes and excessive scarring by blocking the IL-13 pathway. It is believed that dupilumab may help restore the integrity of the nasal epithelium barrier, reducing the thickening and damage seen in chronic inflammation [28]. By blocking the IL-13 pathways, dupilumab can reduce collagen deposition, fibrosis, and abnormal blood vessel formation within nasal polyps. Finally, by controlling inflammation, dupilumab can decrease mucus gland hypertrophy and hyperplasia, leading to reduced mucus production [29]. Clinical studies [7,8] have provided robust evidence demonstrating the efficacy of dupilumab in the treatment of nasal polyps not only in sinonasal symptoms, but also in terms of remodeling with a significant reduction in CT scan opacification. In the SINUS-52 trial [7], treatment with dupilumab led to sustained improvements in CT scan opacification over a one-year period. Furthermore, authors [30] recently demonstrated that dupilumab led to gradual, sustained efficacy on radiological outcomes in the long term.

For all these reasons, treatment with dupilumab can be a valuable alternative to surgery, especially when a revision of frontal surgery is not mandatory because of surgical sequalae. Notably, in our case series, four patients switched from another biologic to dupilumab because of a poor response to the previous biologic anti-type 2 treatments, probably due to a high type 2 inflammatory load. In the absence of treatable traits (smoking, allergens, adherence to INCS, etc.), they were administered dupilumab. In line with the literature data [31], these patients achieved clinically relevant and sustained control over the first year of treatment with dupilumab, confirming that it represents a good option for patients who fail to see sinonasal improvement with other biologics.

The preliminary results of our study should be interpreted in light of some significant limitations, such as the small number of patients. However, it is noteworthy that severe facial pain associated with recurrent frontal sinusitis, without the need for surgery due to complications, is rare. Our data need to be confirmed by multicenter studies involving a greater number of patients. Furthermore, all our patients received previous and heterogenous surgical procedures. In this regard, all patients in our cohort at the time of enrollment had a significant number of previous surgeries but with a low ACCESS score, thus indicating complete or nearly complete surgery. In this context, the condition that could have led to a frontal recurrence is mainly attributable to the high load of type 2 inflammation. Undoubtedly, the evaluation of previous surgery should not be disregarded before starting biological therapy, since the post-surgical anatomical features could preclude the effectiveness of the biologic and predispose the patient to complications. 

## 5. Conclusions

As an anti-type-2 inflammatory pathway biologic agent, our results suggest that the use of dupilumab in patients with recalcitrant frontal sinusitis can lead to a significant improvement of sinonasal symptoms and associated severe cranio-frontal pain, as measured by MIDAS score and VAS craniofacial pain. Furthermore, dupilumab may decrease the need for rescue treatments, including OCS and analgesics, over one year of treatment. No patients required OCS during treatment, and the use of analgesics was drastically reduced.

## Figures and Tables

**Table 1:** Baseline characteristics prior to treatment with dupilumab.

**Demographics**	
Age (mean ± SD; range)	51.4 ± 12.0; 29–67
Female (n/total; %)	6/10; 60%
**Phenotyping**	
Number of previous sinonasal surgeries (mean ± SD)	3.0 + 1.9
ACCESS score (mean ± SD)	2.1 ± 1.7
Time elapsed from last surgery (months; mean ± SD)	34.2 ± 10.5
Concomitant asthma (n/total; %)	8/10; 80%
Peripheral blood hypereosinophilia (n/total; %)	4/10; 40%
NSAID-ERD (n/total; %)	2/10; 20%
Smoking (n/total; %)	1/10; 10%
Brief cycles of OCS in the last year (mean ± SD)	3.6 ± 1.4
Total number of days on OCS in the last year (mean ± SD)	41.8 ± 9.5
NSAID pills/week in the last 2 months (mean ± SD)	9.6 ± 3.1
Previous therapy with a biologic (n/total; %)	4/10; 40%
Omalizumab (n/total; %)	1/4; 25%
Mepolizumab (n/total; %)	2/4; 50%
Benralizumab (n/total; %)	1/4; 25%
Type of previous surgery	
ESS + Draf 2a	3/10; 30%
ESS + Draf 2b	3/10; 30%
ESS + Draf 3	3/10; 30%
ESS + Draf 3 + bicoronal approach	1/10; 10%

Abbreviations: SD, standard deviation; NSAID-ERD, non-steroidal anti-inflammatory drugs–exacerbated respiratory disease; OCS, oral corticosteroids.

**Table 2:** Clinical characteristic of the entire cohort at baseline, in detail.

Case	Age	Number of Previous Surgeries	Type of Previous Surgery	ACCESS Score	Previous Biologics	Brief Cycles of OCS in the Last Year	Total Days of OCS per Year	Time from Last Surgery (Months)	Comorbidities	Mean Number of NSAIDs Pills/Week
1	29	2	FESS, Draf 2b	2	No	5	35	24	Asthma, NSAID-ERD	12
2	63	7	FESS, Draf 2a	0	Oma	6	48	36	Asthma	14
3	66	2	ESS + Draf 3	1	No	4	40	36	Allergic rhinitis	11
4	56	2	ESS+ Draf 3	5	No	2	30	48	Asthma, OSAS	11
5	53	6	ESS + Draf 2b	0	Mepo	5	60	24	Asthma	5
6	62	2	ESS-Draf 3 + external bicoronal approach	3	No	3	45	48	Asthma	7
7	36	2	ESS + Draf 3	4	Benra	4	52	24	Asthma, NSAID-ERD	8
8	45	3	ESS + Draf 2b	3	Mepo	2	32	48	Asthma	11
9	56	2	ESS + Draf 2a	2	No	2	35	26	Allergic rhinitis	12
10	48	2	ESS Draf 2a	1	No	3	41	28	Asthma	5

Abbreviations: ESS, endoscopic sinus surgery; oma, omalizumab; mepo, mepolizumab; benra, benralizumab; OCS, oral corticosteroids; NSAID-ERD, non-steroidal anti-inflammatory drugs–exacerbated respiratory disease.

**Table 3:** Outcomes after 6 months of treatment with dupilumab.

	SNOT-22		NPS		Corticosteroids (Mean Cycles/Year before and after Treatment)		Analgesics (Mean Number or Pills/Week in the Last 2 Months)		MIDAS		VAS Pain		EQ-5D5L	
Case	Pre	Post	Pre	Post	Pre	Post	Pre	Post	Pre	Post	Pre	Post	Pre	Post
1	87	45	2	1	5	0	12	4	60	7	10	5	20	40
2	51	22	0	0	6	0	14	0	55	0	9	1	55	75
3	77	40	6	4	4	0	11	0	42	0	7	0	60	80
4	68	35	2	0	2	0	11	0	48	1	8	1	50	75
5	74	43	4	2	5	0	5	0	26	0	5	1	55	90
6	56	34	5	2	3	0	7	0	30	0	6	1	50	80
7	65	32	4	1	4	0	8	0	51	0	6	1	40	80
8	72	38	3	1	2	0	11	0	45	0	7	0	60	85
9	63	41	3	2	2	0	12	1	46	2	9	0	65	90
10	59	26	2	0	3	0	5	1	53	3	6	2	45	75

Abbreviations: SNOT-22, Sinonasal Outcome Test; NPS, nasal polyp score; VAS, Visual Analogue Scale; MIDAS, Migraine Disability Assessment score.

## Data Availability

The original contributions presented in the study are included in the article; further inquiries can be directed to the corresponding author.

## References

[1] De Corso E., Bellocchi G., De Benedetto M., Lombardo N., Macchi A., Malvezzi L., Motta G., Pagella F., Vicini C., Passali D. (2022). Biologics for Severe Uncontrolled Chronic Rhinosinusitis with Nasal Polyps: A Change Management Approach. Consensus of the Joint Committee of Italian Society of Otorhinolaryngology on Biologics in Rhinology. Acta Otorhinolaryngol. Ital..

[2] De Corso E., Baroni S., Settimi S. (2022). Sinonasal Biomarkers Defining Type 2-High and Type 2-Low Inflammation in Chronic Rhinosinusitis with Nasal Polyps. J. Pers. Med..

[3] Fokkens W.J., Lund V.J., Hopkins C., Hellings P.W., Kern R., Reitsma S. (2020). European Position Paper on Rhinosinusitis and Nasal Polyps 2020. Rhinology.

[4] Zhang L., Zhang Y., Gao Y. (2020). Long-Term Outcomes of Different Endoscopic Sinus Surgery in Recurrent Chronic Rhinosinusitis with Nasal Polyps and Asthma. Rhinology.

[5] DeConde A.S., Mace J.C., Levy J.M. (2017). Prevalence of Polyp Recurrence after Endoscopic Sinus Surgery for Chronic Rhinosinusitis with Nasal Polyposis. Laryngoscope.

[6] Friedman W.H., Katsantonis G.P. (1992). Transantral Revision of Recurrent Maxillary and Ethmoidal Disease Following Functional Intranasal Surgery. Otolaryngol. Head. Neck Surg..

[7] Bachert C., Han J.K., Desrosiers M., Hellings P.W., Amin N., Lee S.E., Mullol J., Greos L.S., Bosso J.V., Laidlaw T.M. (2019). Efficacy and Safety of Dupilumab in Patients with Severe Chronic Rhinosinusitis with Nasal Polyps (LIBERTY NP SINUS-24 and LIBERTY NP SINUS-52): Results from Two Multicentre, Randomised, Double-Blind, Placebo-Controlled, Parallel-Group Phase 3 Trials. Lancet.

[8] De Corso E., Pasquini E., Trimarchi M., La Mantia I., Pagella F., Ottaviano G., Garzaro M., Pipolo C., Torretta S., Seccia V. (2023). Dupilumab in the Treatment of Severe Uncontrolled Chronic Rhinosinusitis with Nasal Polyps (CRSwNP): A Multicentric Observational Phase IV Real-life Study (DUPIREAL). Allergy.

[9] De Corso E., Settimi S., Montuori C., Corbò M., Passali G.C., Porru D.P., Lo Verde S., Spanu C., Penazzi D., Di Bella G.A. (2022). Effectiveness of Dupilumab in the Treatment of Patients with Severe Uncontrolled CRSwNP: A “Real-Life” Observational Study in the First Year of Treatment. J. Clin. Med..

[10] Gallo S., Russo F., Mozzanica F., Preti A., Bandi F., Costantino C., Gera R., Ottaviani F., Castelnuovo P. (2020). Prognostic Value of the Sinonasal Outcome Test 22 (SNOT-22) in Chronic Rhinosinusitis. Acta Otorhinolaryngol. Ital..

[11] Scadding G., Hellings P., Alobid I., Bachert C., Fokkens W., Wijk R.G., Gevaert P., Guilemany J., Kalogjera L., Lund V. (2011). Diagnostic Tools in Rhinology EAACI Position Paper. Clin. Transl. Allergy.

[12] Linder A. (1988). Symptom Scores as Measures of the Severity of Rhinitis. Clin. Exp. Allergy.

[13] Kind P., Hardman G., Leese B. (2005). Measuring Health Status: Information for Primary Care Decision Making. Health Policy.

[14] Passali G.C., Passali D., Cingi C., Ciprandi G. (2022). Smell Impairment in Patients with Chronic Rhinosinusitis: A Real-Life Study. Eur. Arch. Oto-Rhino-Laryngol..

[15] Hummel T., Kobal G., Gudziol H., Mackay-Sim A. (2007). Normative Data for the “Sniffin’ Sticks” Including Tests of Odor Identification, Odor Discrimination, and Olfactory Thresholds: An Upgrade Based on a Group of More than 3000 Subjects. Eur. Arch. Oto-Rhino-Laryngol..

[16] Stewart W.F., Lipton R.B., Dowson A.J., Sawyer J. (2001). Development and Testing of the Migraine Disability Assessment (MIDAS) Questionnaire to Assess Headache-Related Disability. Neurology.

[17] De Corso E., Baroni S., Lucidi D., Battista M., Romanello M., Autilio C., Morelli R., Di Nardo W., Passali G.C., Sergi B. (2015). Nasal Lavage Levels of Granulocyte-Macrophage Colony-Stimulating Factor and Chronic Nasal Hypereosinophilia. Int. Forum Allergy Rhinol..

[18] Masterson L., Tanweer F., Bueser T. (2010). Extensive Endoscopic Sinus Surgery: Does This Reduce the Revision Rate for Nasal Polyposis?. Eur. Arch. Otorhinolaryngol..

[19] Koskinen A., Salo R., Huhtala H. (2016). Factors Affecting Revision Rate of Chronic Rhinosinusitis. Laryngoscope Investig. Otolaryngol..

[20] Watelet J.B., Demetter P., Claeys C., Van Cauwenberge P., Cuvelier C., Bachert C. (2006). Wound Healing after Paranasal Sinus Surgery: Neutrophilic Inflammation Influences the Outcome. Histopathology.

[21] Wormald P., Hoseman W., Callejas C., Weber R.K., Kennedy D.W., Citardi M.J., Senior B.A., Smith T.L., Hwang P.H., Orlandi R.R. (2016). The International Frontal Sinus Anatomy Classification (IFAC) and Classification of the Extent of Endoscopic Frontal Sinus Surgery (EFSS). Int. Forum Allergy Rhinol..

[22] Morrissey D.K., Bassiouni A., Psaltis A.J. (2016). Outcomes of Modified Endoscopic Lothrop in Aspirin-Exacerbated Respiratory Disease with Nasal Polyposis. Int. Forum Allergy Rhinol..

[23] Fischer R., Seebauer C.T., Zeman F., Bohr C., Hosemann W., Weber R., Rohrmeier C., Kuehnel T.S. (2022). Effectiveness of the Lateral Pedicled Endonasal Flap for Prevention of Restenosis in Frontal Sinus Drillouts. Rhin.

[24] Chen F.H., Deng J., Hong H.Y. (2016). Extensive versus Functional Endoscopic Sinus Surgery for Chronic Rhinosinusitis with Nasal Polyps and Asthma: A 1-Year Study. Am. J. Rhinol. Allergy.

[25] Minni A., Dragonetti A., Sciuto A., Rosati D., Cavaliere C., Ralli M., Azimonti D., Franzetti A., de Vincentiis M. (2018). Use of Balloon Catheter Dilation and Steroid-Eluting Stent in Light and Severe Rhinosinusitis of Frontal Sinus: A Multicenter Retrospective Randomized Study. Eur. Rev. Med. Pharmacol. Sci..

[26] Smith T.L., Singh A., Luong A., Ow R.A., Shotts S.D., Sautter N.B., Han J.K., Stambaugh J., Raman A. (2016). Randomized Controlled Trial of a Bioabsorbable Steroid-releasing Implant in the Frontal Sinus Opening. Laryngoscope.

[27] Murr A.H., Smith T.L., Hwang P.H., Bhattacharyya N., Lanier B.J., Stambaugh J.W., Mugglin A.S. (2011). Safety and Efficacy of a Novel Bioabsorbable, Steroid-eluting Sinus Stent. Int. Forum Allergy Rhinol..

[28] Tajiri T., Suzuki M., Nishiyama H., Ozawa Y., Kurokawa R., Takeda N., Ito K., Fukumitsu K., Kanemitsu Y., Mori Y. (2024). Efficacy of Dupilumab for Airway Hypersecretion and Airway Wall Thickening in Patients with Moderate-to-Severe Asthma: A Prospective, Observational Study. Allergol. Int..

[29] Sumi T., Suzuki K., Koshino Y., Ikeda T., Yamada Y., Chiba H. (2024). Successful Treatment of Mucus Plug Due to Allergic Bronchopulmonary Aspergillosis Using Dupilumab. Cureus.

[30] Giombi F., Pace G.M., Nappi E., Giunta G., Muci G., Pirola F., Ferreli F., Heffler E., Paoletti G., Giannitto C. (2024). Radiological Versus Clinical 1-Year Outcomes of Dupilumab in Refractory CRSwNP: A Real-Life Study. Laryngoscope.

[31] Otten J., Van Der Lans R., De Corso E., Dziadziulia K., Hilvering B., Weersink E., Bonini M., Hagemann J., Thaitrakool W., Montuori C. (2023). Evaluation of Switching or Simultaneous Use of Biologic Treatment in Patients with Severe Chronic Rhinosinusitis with Nasal Polyps and Severe Asthma. Considerations in Clinical Decision Making. Expert. Rev. Clin. Immunol..

